# Race Rivalries

**Published:** 1890-08-16

**Authors:** 


### The Doctor's Pulpit.

### RACE RIVALRIES.

The late Mr. Darwin put the thinking world upon a new way of looking at things. Before his time, the soldier had looked with a soldier's eyes upon races civilized and savage; and he had thought out methods for their defeat in the event of war, or for their subjugation and reduction to civilized authority. The lawyer, too, had conceived principles of national and international law. The doctor, following later in the train, had observed how races modify each other by inter-breeding, and how diseases arising in one hemisphere may spread and extend by continuity of vital conditions to the remotest bounds of the world. Still earlier, the mariner and the merchant had encircled and interlaced the globe with an infinity of trade communications. All those classes have been in the habit for centuries of taking more or less cosmopolitan views of men and things. But each class has looked exclusively from its own standpoint. To Mr. Darwin, as to some predecessors and successors of like mind, belongs the rare and distinguishing merit of having observed men of all races as if they were of one race only; of having compared and contrasted them with scientific impartiality; of having noted the facts of their environment; and of having clearly perceived, with an intellectual admiration altogether meritorious, that race rivalries and contests for survival and pre-eminence were the schoolmasters' canes, black boards, and annual prizes, by means of which a gradual ennobling of particular races, and of all men generally, was being surely and decisively effected.

One of the most conspicuous and universal attributes of man is laziness. It may be affirmed in the most positive and absolute way that necessity alone can compel the average man to work. Men will exert themselves in play or in the pursuit of pleasure. But mere exertion is not work. Exertion of a more or less fatiguing kind, carried out for a definite and useful purpose, and persisted in until the purpose is accomplished—that is work; and it is what the undisciplined man of all races instinctively shuns, and often hates. This dislike of work has to be overcome. If we want to see the thing as it presents itself to pure intellect, we must try to put ourselves in the place of the superintending Providence. Looking at the world to-day as a vast school—what single human being is there anywhere who would take the appointment of head master if he had the offer? When we consider the kind of moral and intellectual discipline which the average human being is clearly expected by nature to acquire and to practise, and when we regard the raw material of childhood, youth, ignorance, passion, and prejudice which is the substance into which discipline is to be wrought, and by which it is ultimately to be manifested, there is only one feeling possible to the average human being. That feeling is made up of the two elements of dismay and impossibility.

But complete success attends the tremendous task of enforcing universal labour. Men are induced or compelled by an infinite variety of agencies to set to work and to keep at it. This is a wonderful fact, if we will but think of it for a moment. It is of itself an almost irresistible proof of the existence of an all-commanding divine intellect. "Blind" Nature never could succeed in ruling the human school, and in keeping all the "terrible" pupils at work. One of the most powerful of all agencies in compelling universal industry is that of individual and race rivalry. A little girl known to the writer used to object to bread and milk for breakfast; she preferred bacon and eggs. A small cousin was introduced: and now the little girl makes very determined and generally successful efforts to quite empty her bowl of bread and milk, in order that she may beat her cousin in a daily contest. There is a great deal of human nature in that little girl. Dr. Jones may be a very careless and second-rate practitioner, so long as he reigns in undisturbed possession of the medical throne of Fallowtown. But let Dr. Smith put up his brass plate in the High Street, and Dr. Jones instantly becomes another man in point both of *suaviter in modo* and of *fortiter in re.*

A rival is not a pleasant thought, much less an agreeable reality. The first impulse is to hate him, the second to get rid of him by hook or by crook. The village Russians, for example, are at the present moment suffering themselves to be driven by both these impulses. Many persons honestly believe that the Russians hate the Jews solely or mainly on account of their religion That is a view of the situation which does not commend itself to the scientific mind. The Russians hate the Jews first and chiefly because they are keen competitors and successful rivals in the every-day struggle for existence. Why is it that the Jews are to be expelled from the villages of Russia, whilst they are allowed to pursue their avocations in the towns? The reason, no doubt, is that the village Jew is more successful in farming, or in shopkeeping, or in professional work than his neighbours. Probably he very often puts out his savings at high interest. Thus he becomes a powerful personage, and the imposer of a grievous yoke. Being a member of a small village community, he is soon universally known, and as universally detested. His neighbours hate him, because he is cleverer and more successful than they are, and because his success is at their expense. In response to a clamour which is all but universal the Government resolves to rid the village of him at all costs.

From the standpoint of Mr. Darwin's teaching and of the survival of the fittest this is a blunder of the first order of magnitude. That high philosophic view of the situation is probably one that the Russian villager and his ruling official regard with supreme contempt. But no man with the least pretence to scientific gifts or to practical statesmanship will so regard it. Let it be granted that the Jew is cleverer and more successful than the Russian. It is still possible to make his superiority a positive advantage, even to his village neighbours. His presence as a rival and competitor affords a constant stimulus to intellectual activity and business effort which is of the greatest possible value. If it be the fact that the successful Jew acts unfairly and oppressively by his Christian neighbours, that may be a reason for imposing certain restraints upon his business license, but it cannot be a reason for expelling him root and branch from the soil. It is not denied that he is a loyal subject and an industrious and useful citizen. If, in addition to being loyal and industrious, he is also a constant and powerful stimulus to intellectual effort and business capacity, he must be regarded from the standpoint of science and true statesmanship as one of the most valuable of all the factors that tend to promote national development.

We have not yet reached a period in which the average man is capable of observing for himself the action of those general laws which the scientist has perceived in nature and formulated in terms of human speech. Still less does the ordinary citizen take trouble to encourage and promote the success of those race factors which make for the highest development of his kind. Every man thinks he does enough for humanity when he fights for his own hand. But an intelligent science and a worthy religion agree in affirming this—that he is the most successful fighter for his own hand who admits to the full the right of his neighbours to fight on equal terms with himself. Race rivalry is in every way advantageous to all the races which engage in the same great life contest. Race hatred is one of the maddest, stupidest, and wickedest feelings in which any man or people can indulge. In this country there are persons who hate the Jew, the German, the Russian, and the Frenchman equally, and on the common ground that they make by their rivalry the battle of life more difficult to win. It is an insane hatred; and, like all insanities, needs to be put under firm restraint. Science affirms the unity of the human race; religion proclaims its common divine paternity; reason and right feeling accept the truth, and strive to bring into brotherhood all the races of men on the face of the earth.

